# The Impact of Nonconvulsive Status Epilepticus after Cardiac Surgery on Outcome

**DOI:** 10.3390/jcm11195668

**Published:** 2022-09-26

**Authors:** Keso Skhirtladze-Dworschak, Alessia Felli, Susanne Aull-Watschinger, Rebekka Jung, Mohamed Mouhieddine, Andreas Zuckermann, Edda Tschernko, Martin Dworschak, Ekaterina Pataraia

**Affiliations:** 1 Department of Anesthesia, Intensive Care Medicine and Pain Medicine, Division of Cardiac Thoracic Vascular Anesthesia and Intensive Care Medicine, General Hospital Vienna, Medical University of Vienna, Waehringer Guertel 18-20, A-1090 Vienna, Austria; 2 Department of Anesthesia, Intensive Care Medicine and Pain Medicine, Division of General Anesthesia and Intensive Care Medicine, Medical University of Vienna, A-1090 Vienna, Austria; 3 Department of Neurology, Medical University of Vienna, A-1090 Vienna, Austria; 4 Department of Cardiac Surgery, Medical University of Vienna, A-1090 Vienna, Austria

**Keywords:** cardiac surgery, cardiopulmonary bypass, brain insult, nonconvulsive status epilepticus, conventional electroencephalography, seizures, outcome

## Abstract

Neurological complications after heart surgery are associated with tremendous morbidity and mortality. Nonconvulsive status epilepticus (NCSE), which can only be verified by EEG, may cause secondary brain damage. Its frequency and its impact on outcomes after cardiac surgery is still unclear. We collected the neurological files and clinical data of all our patients after heart surgery who, in the course of their ICU stay, had been seen by a neurologist who ordered an EEG. Within 18 months, 1457 patients had cardiac surgery on cardiopulmonary bypass. EEG was requested for 89 patients. Seizures were detected in 39 patients and NCSE was detected in 11 patients. Open heart surgery was performed in all 11 NSCE patients, of whom eight showed concomitant brain insults. None had a history of epilepsy. Despite the inhibition of seizure activity with antiseizure medication, clinical improvement was only noted in seven NCSE patients, three of whom were in cerebral performance category 2 and four in category 3 at hospital discharge. The four patients without neurological benefit subsequently died in the ICU. The occurrence of NCSE after open cardiac surgery is significant and frequently associated with brain injury. It seems prudent to perform EEG studies early to interrupt seizure activity and mitigate secondary cerebral injury.

## 1. Introduction

Despite improvements in perioperative care, the availability of new neuromonitoring tools and more refined surgical techniques, the impact of neurological complications after cardiac surgery is still substantial. They are associated with impaired perioperative and long-term outcome and a lower quality of life. Clinical manifestations vary and can include short-lasting cognitive-behavioral disturbances, seizures and severe brain injury [1]. The incidence of postoperative seizures after cardiac surgery has been estimated to range between 0.5% and 9% and reported recurrence rates are around 40–66% [2,3,4]. The temporal threshold that defines an abnormally prolonged seizure is five minutes for convulsive status epilepticus. For nonconvulsive status epilepticus (NCSE), i.e., status epilepticus without prominent motor symptoms, the threshold is 10 min. Not included in these definitions, but still widely used by clinicians, are instances in which brief intermittent seizure activity occurs without convulsions, with no full recovery of consciousness between attacks [5,6]. In patients with baseline coma or encephalopathy, NCSE patients typically show more than 30 total minutes of ictal EEG activity at any given hour of recording (i.e., >50 percent of the record). Due to its subtle symptoms, NCSE can easily be missed or misidentified and can only be verified via an EEG, resulting in frequent undertreatment [5]. The overall rate of occurrence of NCSE in critically ill patients lies somewhere between 8% and 40% whereupon NCSE can contribute to secondary brain injury [7,8]. Status epilepticus in general is a risk factor for poor outcomes after focal or global brain ischemia, which is not uncommon in heart surgery [9]. However, there is an ongoing debate regarding whether seizures are independently associated with mortality in intensive care unit (ICU) patients and in those patients with altered consciousness [10,11]. In the cardiac surgical population, where neurological complications as a cause for morbidity and mortality come in second after cardiovascular dysfunction, the true incidence of NCSE and its impact on outcomes is still unknown [2]. Therefore, the aim of this retrospective study was to evaluate the frequency of NCSE after adult cardiac surgery on cardiopulmonary bypass (CPB) and to describe specific characteristics, as well as clinical outcomes, of affected patients.

## 2. Methods

### 2.1. Study Design

This retrospective study was performed at the Division of Cardiac Thoracic Vascular Anesthesia and Intensive Care Medicine at the General Hospital Vienna. The study protocol has been approved by the local institutional Ethics Committee (Study Protocol 1620/2016 and 1157/2018). Due to the retrospective nature of the study and the state-of-the-art treatment patients received, the Ethics Committee waived the need to obtain informed consent. Reporting was performed in accordance with the STROBE guidelines (http://www.strobe-statement.org (accessed on 21 September 2022)).

All patients who underwent heart operations with CPB between 1 January 2014 and 30 June 2015 were screened for allocation to the Department of Neurology and a subsequent EEG examination (this is hereafter termed the “EEG group”). Reasons for this assignment were new, noticeable neurological problems likely related to seizures (i.e., vigilance disorders, abnormal movements such as myoclonus or oral automatisms, presumed seizure activity, confusion, or inadequate wake-up reactions despite no or minimal sedation). The EEGs were performed with a commercially available digital video-EEG system (Alpha-Trace 32-channel EEG record, B.E.S.T Medical Systems, Austria) for at least 30 min with EEG electrodes placed according to the extended 10–20 system with additional fronto-temporal electrodes (FT9/FT10). If the EEG fulfilled the criteria of NCSE, the videos were screened to determine clinical correlates. The recently validated Salzburg criteria were applied for the diagnosis of status epilepticus [12]. All EEG recordings had previously been reviewed by two EEG-board certified authors (E.P. and S.A.-W.) and the neurological files, as well as the corresponding clinical data, were again checked for proper diagnosis and completeness in the course of the retrospective evaluation.

In addition, a neurologically asymptomatic “control group”, composed of patients who likewise underwent cardiac surgery on CPB but exhibited no new neurological symptoms postoperatively, was matched according to age, gender, body weight and type of intervention in order to identify potential predictors of postoperative complications most probably related to seizures and particular specifics of these patients.

### 2.2. Intraoperative Management

All patients received general anesthesia for their heart surgery that was performed on CPB. The surgical technique varied according to individual preferences.

For induction of general anesthesia patients received midazolam (0.04 mg/kg), propofol (1.0–1.5 mg/kg), fentanyl (3–10 µg/kg) and cis-atracurium (0.2 mg/kg). Anesthesia was maintained with sevoflurane (target BIS value: 40–50) and a continuous fentanyl infusion (3–5 µg/kg/h). After insertion of a central venous catheter, all patients underwent a transesophageal echocardiography (TEE) examination. TEE was used (1) to assess myocardial performance, (2) to evaluate the function of heart valves, (3) to determine intracardiac shunts and (4) to identify calcifications within the thoracic aorta. The site of aortic cannulation and cross clamping was chosen according to a preoperatively performed CT scan of the thorax in all elective cases. Patients with normal kidney function received tranexamic acid (10 mg/kg) as an antifibrinolytic agent before the start of CPB and the same dose in the CPB prime. Patients with impaired kidney function and serum creatinine levels > 1.5 mg/dL received a reduced dosage, given either as infusion or added to the pump prime. After anticoagulation with heparin (400 IE/kg IV) and achieving an activated clotting time > 400 s, CPB was started using non-pulsatile flow at 2.3–2.7 L/min/m^2^, a non-heparin-coated circuit and a membrane oxygenator (Quadrox^TM^, Maquet, Hirrlingen, Germany, or Dideco Compact Flow TM, Mirandola, Italy). Priming of the CPB circuit varied according to the surgeons’ preferences. It could either be primed with Ringer’s Lactate alone or in combination with a colloid solution (e.g., Volulyt^®^ 6% or Human Serum Albumin 5%). The ascending aorta was the preferred site for arterial cannulation. In patients with aortic calcifications or ascending aortic dissection and in patients undergoing reoperations, the right subclavian artery was favored for arterial cannulation via a sewn-in Dacron graft. Mild-to-moderate hypothermia on CPB varied between 32 °C and 35 °C, according to the surgeons’ preferences. Deep hypothermia was applied in cases that required circulatory arrest. Carbon dioxide insufflation into the operative field was routinely used during open-chamber heart surgery. De-airing of the bypass circuit and the heart chambers after surgery was performed according to standard technique and the latter was controlled via TEE. After the completion of surgery, all patients were transferred to an intensive care unit (ICU) for further observation and treatment.

### 2.3. Statistical Analysis

All patient demographics, their past medical and neurological history, as well as their perioperative data, were collected retrospectively. Quantitative variables were analyzed using Pearson’s chi-squared test and depicted as absolute numbers. Qualitative variables were analyzed with a *t*-test for normally distributed variables and the Wilcoxon rank-sum test for non-normally distributed variables. Variables with *p* < 0.15 were included in a multivariable logistic regression analysis. Logistic regression analysis was used to predict independent risk factors for postoperative neurological complications and the occurrence of NCSE. Normally distributed variables are expressed as mean ± standard deviation and non-normally distributed variables as median and range. A *p*-value of <0.05 was considered statistically significant. Statistical analysis was carried out using Stata^®^ 15.1 (StataCorp LLC, College Station, TX, USA).

## 3. Results

Between 1 January 2014 and 30 June 2015, one thousand four hundred fifty-seven adult patients underwent heart surgery performed with CPB. Eighty-nine of them exhibited neurological symptoms most likely related to seizures and were examined by a consultant neurologist, who consecutively assigned them to an EEG study. These patients formed the “EEG group”. The examinations were exclusively assessed by board-certified electrophysiologists and epileptologists.

**Figure 1:**
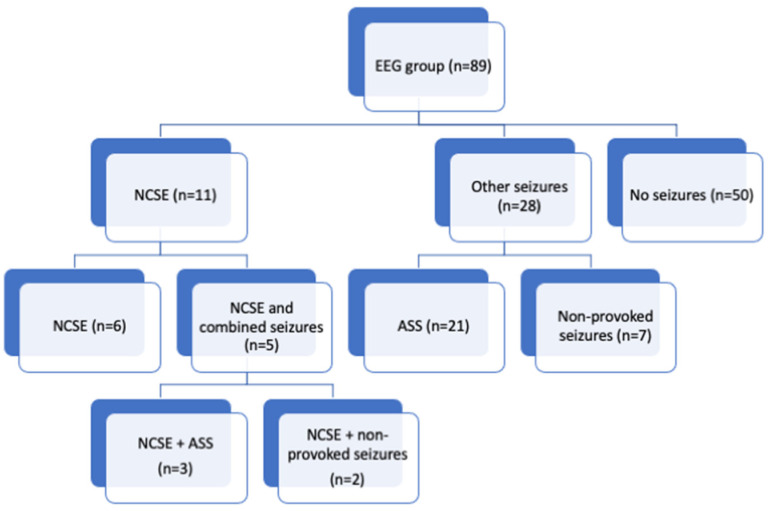
Frequency of seizures in patients undergoing EEG studies. NCSE, patients with clear ictal activity in their EEG in accordance with the Salzburg criteria (see [12]); Other seizures, distinct clinically observed seizures in the ICU with or without ictal EEG activity at the time of the EEG study; No seizures, altered consciousness ± abnormal movements but no evident clinically observed seizures or corresponding ictal EEG changes.

Of the 89 patients, 39 subjects (3% of the total cohort, consisting of 1457 patients at risk) had clinically observed seizures and/or ictal activity verified via EEG. The clinical manifestations that initiated an EEG study in the 50 patients without confirmed seizures were related to unspecific non-epileptic movements or oral automatisms, prolonged actions of anesthetics or opioids or symptoms associated with cerebral embolic or hemorrhagic injury. Eleven patients (0.8%) developed NCSE. Six of them showed isolated NCSE, whereas five patients had foregone clinically observed seizures, either in the form of acute symptomatic seizures (ASS), occurring within the first 7 days after a specific insult, or unprovoked seizures. Another twenty-one patients from the EEG group exhibited only ASS and seven patients developed non-provoked seizures further along the line (Figure 1). In the EEG cohort comprising 89 patients, none had psychogenic or any other kind of non-epileptic seizures. Within the EEG group there was no statistically significant difference between patients with or without NCSE in regard to demographic, perioperative, and outcome data. Perioperative cerebrovascular insults were frequently seen in patients with NCSE. Cranial computed tomography (CCT) documented new ischemic stroke or hemorrhage in eight of 11 patients with NCSE (57%), whereas CCT results demonstrated insults in 6 of 28 patients with other types of seizures (21%) and in 14 of 50 patients without seizures (28%, *p* = 0.01). Ischemic strokes were observed in 2 NCSE, 2 NCSE + ASS, 3 ASS and 13 patients with no seizures, whereas hemorrhagic insults could be verified in 1 NCSE, 1 NSCE + non-provoked seizures and 3 non-provoked seizures patients, as well as in 1 patient without seizures. Ischemic lesions next to hemorrhagic insults were detected in the CT scans of two patients with isolated NCSE.

Details regarding the surgical procedures performed are given in Table 1. All 11 patients with NCSE had undergone open chamber cardiac surgery. Seven patients had combined (two or more) procedures. One patient required aortic valve replacement following acute endocarditis. Another patient underwent mitral valve replacement for mitral insufficiency, one patient was undergoing heart transplantation (HTX) and another patient received a left ventricular assist device (LVAD). No patient, except one, had a history of previous neurological accidents. This particular patient already presented with a right-hemispheric media infarction on admission and was in hemorrhagic and septic shock due to endocarditis of the aortic valve, complicated by gastrointestinal bleeding and acute kidney insufficiency (Table 2).

As mentioned above, patients with neurologically conspicuous symptoms presumably related to seizure activity formed the EEG group. Comparison between this EEG group (n = 89) and the neurologically intact control group (n = 92) revealed the following. Significantly more patients in the EEG group suffered from cerebral arterial occlusive disease, extensive aortic calcification and chronic or acute kidney insufficiency (Table 1). Twenty-four patients from the EEG group underwent re-operative cardiac surgery, compared to 14 patients from the control group (*p* = 0.052). Nineteen patients (21%) from the EEG group had been resuscitated perioperatively, whereas this was only the case in one patient (1%) from the control group (*p* < 0.05). Furthermore, the duration of anesthesia, intensive care and the hospital length of stay were significantly prolonged in patients from the EEG group. In-hospital mortality was also significantly higher in the EEG group (*p* < 0.00001). Within the EEG group, 65 patients survived to hospital discharge, whereas 24 patients died during their hospital stay. Four of them exhibited NCSE and 20 patients exhibited bilateral tonic-clonic seizures after surgery. The most frequent causes of death were either sepsis with multiple organ failure (11 patients), cardiac decompensation (7 patients) or multiple cerebral embolic and hemorrhagic insults leading to major irreversible brain damage (6 patients). No patients died from prolonged NCSE. Antiseizure medication led to the abortion of NCSE in all patients. The majority of the seven NCSE patients who survived until hospital discharge had poor functional outcomes. Four showed severe (cerebral performance category; CPC 3) and three showed moderate cerebral disability (CPC 2). The four patients who died in the ICU never regained consciousness. Consequently, their CPC scores could not be determined. The average duration of anesthesia in the NCSE subgroup was 467 min, whereas the durations of CPB and aortic cross clamp time were 194 and 105 min, respectively. Three of the 11 NCSE patients had required urgent surgery and none of these patients had a previous history of seizures. Details of the 11 NCSE patients are given in Table 2. Owing to the small group size, we could not determine any independent risk factor for the development of NCSE through logistic regression analysis.

## 4. Discussion

To our knowledge, this is the first large-scale study to date that specifically investigated the frequency of postoperative de novo NCSE and its specific outcomes in cardiac surgery patients operated with CPB. During the observation period, 89 (i.e., 6%) of all included patients (n = 1457) had developed some kind of neurological dysfunction in the ICU that warranted subsequent EEG studies. Thirty-nine of these patients (i.e., 3% of the total cohort and 44% of the EEG group) had clinically evident seizures and/or ictal EEG activity, which is in line with previously published rates in the adult population [3,13]. The majority of affected patients (i.e., 24 patients) exhibited ASS, i.e., clinical seizures in close temporal association with a documented cerebral insult, which could be any injurious event related to—but not exclusively to—exposure to heart surgery with extracorporeal circulation. ASS was also present in three of the 11 patients with NCSE. In another two patients, NCSE was associated with delayed unprovoked seizures, whereas six NCSE patients showed no further clinically evident seizure activity. This particular subgroup, however, could potentially be larger, as some especially short-lasting episodes of NCSE could have been missed due the absence of characteristic symptoms or could have escaped detection in serial EEG studies [2]. Continuous EEG (cEEG) recording has therefore been advocated for the purpose of diagnosing NCSE in an ICU setting [14,15,16]. Unfortunately, cEEG is not available in every institution, it is expensive, the detailed review of the recordings and its interpretation can be time-consuming and it can potentially be less reliable, particularly when a reduced electrode array is employed [17]. Additionally, its sensitivity in detecting NCSE is much lower than that of a conventional EEG, particularly when EEG strips are analyzed retrospectively without video recordings of the patients [4,13]. Although cEEG has been associated with seizures being detected more often and with more frequent adjustments of antiseizure treatment, routine EEG was not found to be inferior in regard to the reduction of 6-month mortality as compared to cEEG [11].

In this study, neurological complications potentially associated with seizure activity after cardiac surgery on CPB were also related with re-operative surgery, urgent and open-chamber procedures, renal insufficiency, cerebrovascular disease, aortic atherosclerosis, cardiopulmonary resuscitation and a prolonged duration of anesthesia causing tremendously increased morbidity and mortality [9,18,19]. In this cohort, seizures were closely linked to postoperative mortality signifying the multiple co-morbidities of these patients and the severity of the critical state these patients were in. Cerebrovascular insults in this investigation were often associated with convulsive and non-convulsive seizure activity, whereby in this study embolic stroke in the context of open-heart surgery was the most frequent underlying lesion in NCSE patients, followed by cerebral hemorrhage. Structural brain injury could be confirmed in eight of the 11 NCSE patients in CCTs performed after surgery. Had we conducted MRI studies, we would probably have detected more insults due to their greater sensitivity in comparison to CCT. Such investigations, however, were not feasible for most of our patients. Four of the NCSE patients subsequently died in the course of their hospital stay without showing any clinical improvement following the initiation of appropriate antiseizure medication despite the abolishment of the seizure activity [20]. Cerebral injury also accounts for the 62% mortality rate we found in patients exhibiting any type of seizure postoperatively and replicates findings by other researchers who showed that poor outcomes were primarily seen in seizure patients who concomitantly presented with new brain injury [3,8,18]. Even if there is no visible structural damage detected in the CCT images, subtle neuronal insults may occur in the context of CPB [21,22,23], which cause encephalopathy and might also induce epileptic activity in susceptible patients [24,25]. Especially when cerebral damage has already manifested itself, it becomes even more important to detect NCSE early, so that it can be treated quickly and vigorously in order to prevent additional neuronal injuries, thereby reducing severe disability [26,27]. However, similarly to patients with aneurysmal subarachnoid hemorrhage, greater success could potentially be achieved in patients without predominant structural brain damage, in whom concomitant NCSE had a more extensive impact on unfavorable outcomes [28]. The failure of appropriate antiseizure treatment to lead to clinical improvement and NCSE associated with major structural brain damage could further indicate poor outcomes.

### Limitations

This was a retrospective single center study, which limits the generalizability of the results. Furthermore, we cannot eliminate potential confounders and may not have accounted for more recent changes in patient management. On the other hand, for the chosen time period, we had exclusive access to the complete set of ICU data, the results of all the neurological evaluations and the total EEG recordings for all our patients. This allowed us to obtain an all-encompassing picture of the actual clinical situation. As has already been mentioned above, despite the mandatory daily clinical neurological assessments by intensivists, the true incidence of NCSE could still be somewhat higher since (1) suspicious events might not have drawn the attention of the nurses and the physicians in charge, (2) they could have been misinterpreted by the caregivers and (3) their detection may have been skipped due to the intermittent nature of the EEG recording. We can further not completely exclude that certain patients from the control group had also been assigned to a neurologist. However, we can definitively rule out the notion that these patients demonstrated any clinical signs which could be explained by seizure activity that led to such a consultation. In order to determine potential risk factors for postoperative neurological complications that are likely associated with seizures and, at the same time, eliminating major confounders (e.g., age, gender, body weight and type of intervention), we compared the EEG group with control patients who most likely had not been affected by any seizure activity after surgery. However, the number of patients suffering from NCSE did not allow us to conduct further, more detailed statistical analyses to elucidate specific determinants for NCSE. Multi-center studies may overcome this handicap by increasing the number of patients at risk who can be evaluated. Unfortunately, such studies often concomitantly have the inherent flaw of including quite heterogenous populations and participating institutions, which may obscure the clinical significance of findings that are pertinent to certain subpopulations (such as the one we investigated at a tertiary care center managing complex cases and many high-risk patients). This may eventually necessitate the screening of an even larger group of patients in order to obtain significant results for this particular subgroup. As cardiac surgical units can markedly differ in their caseloads and their respective patient management approaches, the overall findings of studies which include a variety of centers may therefore be limited to only some of them, and may not be applicable to every center. The participation of a great number of centers may additionally impede strict protocol compliance, which can further confound the obtained results. We thus believe that our observations from a single tertiary care center with standardized patient management protocols and the detailed information that was available to us provide a good insight in the occurrence of NCSE in this patient population, reveal potential causative factors, and highlight the poor outcomes of affected cardiac surgical patients.

## 5. Conclusions

In summary, the frequency of NCSE in this large cohort of patients who had undergone cardiac surgery on CPB was 0.8%. NCSE was frequently associated with structural brain damage, as well as poor outcomes. Since NCSE can independently lead to secondary brain injury, it nevertheless seems prudent to evaluate suspicious patients via an EEG study at an early stage to adequately treat and disrupt NCSE. This approach should positively affect early mortality in patients without major comorbidities [20]. If the focus lies on patients as described in the Methods section (i.e., the composition of our EEG group), the chance of detecting seizure activity in the respective EEGs will be almost 50%, which will narrow down the use of scarce resources to a relatively small and manageable subset of patients (in our case, 89 of 1457 patients) treated in the ICU after cardiac surgery with CPB. In this study, corresponding antiseizure treatment abrogated previously confirmed seizure activity in all NCSE patients but only resulted in clinical improvement in survivors. It therefore remains unclear whether the rapid detection of NSCE will significantly impact morbidity and mortality in patients with severe brain insults. It might, however, aid in prognostication.

## Figures and Tables

**Table 1:** Characteristics, intraoperative details and outcome data from patients with subsequent EEG studies and from neurologically asymptomatic patients.

Variable	EEG Group (n = 89)	Asymptomatic Control Group (n = 92)	*p* Value
**Preoperative characteristics**			
Age, (years)	69 ± 12	68 ± 11	0.307
Gender, M/F, (n)	58/31	61/31	0.872
Body weight, (kg)	78 ± 16	80 ± 16	0.485
Height, (cm)	171 ± 10	171 ± 15	0.923
BMI, (kg/m^2^)	27 ± 4	27 ± 4	0.647
Prior myocardial infarction, (n)	24	21	0.519
No sinus rhythms, (n)	36	29	0.211
Prior cardiac surgery, (n)	24	14	0.052
Implanted PM/ICD, (n)	21	15	0.219
Left ventricular ejection fraction, (n) <30% 30–50% >50%	25 20 44	20 22 50	0.611
Diabetes mellitus, (n)	25	20	0.323
Acute or chronic kidney injury, (n)	40	22	0.003
Chronic obstructive pulmonary disease, (n)	21	14	0.154
Peripheral arterial occlusive disease, (n)	17	14	0.488
Cerebral arterial occlusive disease, (n)	17	5	0.005
Endocarditis, (n)	5	2	0.694
Previous cerebrovascular accident, (n)	15	13	0.257
History of seizures (n)	3	0	
Extensive aortic calcification or atherosclerosis, (n)	14	2	0.003
**Intraoperative details**			
Urgent or emergent operation, (n)	22	25	0.706
Surgical Procedure, (n)			0.114
CABG	16	17	
Other valve operations	7	7	
Aortic valve replacement	15	15	
Ascending aortic surgery	7	8	
LVAD	7	8	
HTX	7	7	
Combined procedures	30	30	
Duration of anesthesia (min)	439 (175–980)	398 (222–699)	0.036
Duration of surgery (min)	340 (125–945)	304 (160–597)	0.108
Duration of CPB (min)	158 (30–505)	149 (50–322)	0.307
Duration of ACC (min)	94 (23–300)	91 (27–201)	0.942
Deep hypothermic circulatory arrest, (n)	10	8	0.568
Tranexamic acid, (g)	1.4 (0.4–3.0)	1.4 (0.5–3.0)	0.361
Intraoperative fluid balance, (ml)	5050 (884–20,169)	4496 (310–17,799)	0.859
Use of vasopressors, (%) Low High	95 6 94	91 49 51	0.256
Transfusion of packed red blood cells, (mL)	900 (300–4500)	600 (250–4800)	0.077
Transfusion of fresh frozen plasma, (mL)	800 (200–4600)	800 (128–4000)	0.706
Transfusion of platelets, (mL)	245 (104–719)	193 (100–1000)	0.415
**Postoperative data**			
ICU length of stay, (days)	32 ± 33 22 (1–160)	9 ± 22 2 (1–175)	0.00001
Hospital length of stay, (days)	57 ± 48 43 (8–270)	26 ± 26 16 (8–178)	0.00001
Mortality, (n) 30-day In-hospital	3 24	1 1	0.296 0.00001
CPR perioperatively, (n)	19	1	0.0002
Postoperative stroke, (n)	28	0	<0.05
Postoperative seizures, (n)	39	0	<0.05

BMI, body mass index; PM, pacemaker; ICD, internal cardioverter/defibrillator; CABG, coronary artery bypass graft surgery; LVAD, left ventricular assist device; HTX, heart transplantation; CPB, cardiopulmonary bypass; ACC, aortic cross clamp; ICU, intensive care unit; CPR, cardiopulmonary resuscitation.

**Table 2:** Perioperative data from patients with confirmed de novo NCSE after cardiac surgery on cardiopulmonary bypass.

Patient	1	2	3	4	5	6	7	8	9	10	11
Age	75	41	68	63	72	88	74	84	85	68	84
Gender	F	M	M	M	M	M	M	F	F	F	F
BMI	26	25	26	25	26	25	23	22	21	21	27
LVEF (%)	31–50	>50	<30	<30	>50	<30	>50	>50	31–50	>50	>50
Reoperation	Yes	No	No	Yes	No	No	No	No	No	No	No
Severe aortic calcifications	Yes	No	No	No	No	No	No	Yes	No	No	Yes
CVOD	No	No	No	Yes	No	No	Yes	No	Yes	No	No
Previous neurological insult	No	Yes	No	No	No	No	Yes (TIA)	Yes	No	No	No
AKI/CKI	Yes	Yes	Yes	No	No	Yes	No	No	No	No	No
Surgery	AVR/CABG	AVR	HTX	LVAD	MVR	AVR/CABG	AVR/TVR/CABG	AVR/CABG	CABG/MVR/TVR	MVR/TVR	Bio-Bentall
CPBT (min)	212	137	277	134	125	171	324	111	184	217	237
ACCT (min)	157	49	75	0	97	135	166	81	109	140	150
Surgery time (min)	401	232	535	305	200	406	409	289	342	315	510
Duration of anesthesia (min)	506	320	655	457	252	536	596	372	418	437	580
Urgency of procedure	Urgent	Urgent	Urgent	Elective	Elective	Elective	Elective	Elective	Elective	Elective	Elective
Known CVA or seizures	No/No	Yes/No	No/No	No/No	No/No	No/No	TIA/No	Yes/No	No/No	No/No	No/No
Conscious-ness, neurological symptoms	Neurological deterioration somnolence, motor restlessness	Neurolo-gical deterio-ration, coma	Somnolence, coma, peri-oral myoclonus	Grand-mal seizures	Awake, myoclonus	Coma, repetitive head movements	Coma, suspected seizures	Awake, grand-mal seizures, mono-paresis of left arm	Awake, hemiparesis left side	Coma, myoclonus	Sedated, tonic-clonic seizures
Neuro-imaging CCT	Subacute SAB and micro-bleeds in the left fronto-parietal lobe	Media infarction with hemor-rhagic transfor-mation in the right hemisphere	Right temporal ischemic insult	SAB	No pathology	No pathology	Ischemic stroke	Ischemic and hemor-rhagic stroke	No pathology	Right peri-ventricular insult	Brain stem infarction
CCT POD	12	preop. CCT	6	25	19	9	2	9	2	1	1
EEG POD	14	26	26	27	20	13	2	11	3	1	1
EEG finding	NCSE	NCSE	NCSE	NCSE + EPI	NCSE + EPI	NCSE	NCSE	NCSE	NCSE + ASS	NCSE + ASS	NCSE + ASS
Treatment	Phenytoin Levetira-cetam Lorazepam	Phenytoin Levetira-cetam Lorazepam	Levetira-cetam	Levetira-cetam Lorazepam	Levetira-cetam	Phenytoin Levetira-cetam	Phenytoin Levetira-cetam	Phenytoin Levetira-cetam Lorazepam	Levetira-cetam Lacosamide	Levetira-cetam Lacosamide	Levetira-cetam
Improvement in EEG	Yes	Yes	Yes	Yes	Yes	Yes	Yes	Yes	Yes	Yes	Yes
Improvement of general condition	No	No	No	No	Yes (slowly)	Yes (slowly)	Yes (slowly)	Yes	Yes	Yes	Yes
ICU stay (d)	37	65	49	112	73	42	92	7	21	5	10
Hospital stay (d)	63	65	49	235	103	54	172	8	23	11	56
Death/day after surgery	Yes/176	Yes/161	Yes/149	Yes/190	No	No	No	No	No	No	No
CPC at hospital discharge	N/A	N/A	N/A	N/A	CPC 2	CPC 3	CPC 3	CPC 2	CPC 3	CPC 2	CPC 3

NCSE, non-convulsive status epilepticus; AVR, aortic valve replacement; CABG, coronary artery bypass surgery; HTX, heart transplantation; LVAD, left ventricular assist device; MVR, mitral valve replacement; TVR, tricuspid valve replacement/reconstruction; CPBT, cardiopulmonary bypass time; ACCT, aorta cross clamp time; CVA, cerebrovascular accident; TIA, transitory ischemic attack; CCT, cranial computerized tomography; SAB, subarachnoid hemorrhage; EEG, electroencephalogram; EPI, non-provoked seizures; POD, postoperative day; ICU, intensive care unit; d, days; LVEF, left ventricular ejection fraction; CVOD, cerebrovascular occlusive disease; AKI, acute kidney injury; CKI, chronic kidney injury; CPC, cerebral performance category; N/A, not available.

## Data Availability

The data that support the findings of this study are available from the corresponding author upon reasonable request.

## Abbreviations

AVR, aortic valve replacement; ACCT, aortic cross clamp time; AKI, acute kidney injury; ASS, acute symptomatic seizures; CABG, coronary artery bypass graft surgery; CCT, cranial computerized tomography; cEEG, continuous electroencephalogram; CKI, chronic kidney injury; CPBT, cardiopulmonary bypass time; CPC, cerebral performance category; CPR, cardiopulmonary resuscitation; CVA, cerebrovascular accident; CVOD, cerebrovascular occlusive disease; EEG, electroencephalogram; FT, fronto-temporal electrodes; HTX, heart transplantation; LVAD, left ventricular assist device; LVEF, left ventricular ejection fraction; MVR, mitral valve replacement; NCSE, non-convulsive status epilepticus; POD, postoperative day; ICU, intensive care unit; SAB, subarachnoid hemorrhage; TIA, transitory ischemic attack; TVR, tricuspid valve replacement/reconstruction.
